# The Reliability and Predictive Ability of a Biomarker of Oxidative DNA Damage on Functional Outcomes after Stroke Rehabilitation

**DOI:** 10.3390/ijms15046504

**Published:** 2014-04-16

**Authors:** Yu-Wei Hsieh, Keh-Chung Lin, Mallikarjuna Korivi, Tsong-Hai Lee, Ching-Yi Wu, Kuen-Yuh Wu

**Affiliations:** 1 Department of Occupational Therapy and Graduate Institute of Behavioral Sciences, College of Medicine, Chang Gung University, Taoyuan 333, Taiwan; E-Mails: ywhsieh@mail.cgu.edu.tw (Y.-W.H.); mallik.k5@gmail.com (M.K.); 2 Healthy Aging Research Center, Chang Gung University, Taoyuan 333, Taiwan; 3 School of Occupational Therapy, College of Medicine, National Taiwan University, Taipei 100, Taiwan; E-Mail: kehchunglin@ntu.edu.tw; 4 Division of Occupational Therapy, Department of Physical Medicine and Rehabilitation, National Taiwan University Hospital, Taipei 100, Taiwan; 5 Stroke Center and Department of Neurology, Chang Gung Memorial Hospital, Linkou Medical Center and College of Medicine, Chang Gung University, Taoyuan 333, Taiwan; E-Mail: thlee@adm.cgmh.org.tw; 6 Institute of Occupational Medicine and Industrial Hygiene, National Taiwan University, Taipei 100, Taiwan; E-Mail: kuenyuhwu@ntu.edu.tw

**Keywords:** oxidative stress, stroke, rehabilitation, biomarker, 8-OHdG, clinical evaluation

## Abstract

We evaluated the reliability of 8-hydroxy-2′-deoxyguanosine (8-OHdG), and determined its ability to predict functional outcomes in stroke survivors. The rehabilitation effect on 8-OHdG and functional outcomes were also assessed. Sixty-one stroke patients received a 4-week rehabilitation. Urinary 8-OHdG levels were determined by liquid chromatography–tandem mass spectrometry. The test-retest reliability of 8-OHdG was good (interclass correlation coefficient = 0.76). Upper-limb motor function and muscle power determined by the Fugl-Meyer Assessment (FMA) and Medical Research Council (MRC) scales before rehabilitation showed significant negative correlation with 8-OHdG (*r* = −0.38, *r* = −0.30; *p* < 0.05). After rehabilitation, we found a fair and significant correlation between 8-OHdG and FMA (*r* = −0.34) and 8-OHdG and pain (*r* = 0.26, *p* < 0.05). Baseline 8-OHdG was significantly correlated with post-treatment FMA, MRC, and pain scores (*r* = −0.34, −0.31, and 0.25; *p* < 0.05), indicating its ability to predict functional outcomes. 8-OHdG levels were significantly decreased, and functional outcomes were improved after rehabilitation. The exploratory study findings conclude that 8-OHdG is a reliable and promising biomarker of oxidative stress and could be a valid predictor of functional outcomes in patients. Monitoring of behavioral indicators along with biomarkers may have crucial benefits in translational stroke research.

## 1. Introduction

Biomarkers of oxidative stress are defined as a biological molecule whose chemical structure has been modified by free radicals [1]. Stroke survivors produce more free radicals and are vulnerable to oxidative stress; therefore, measuring the stress biomarkers in stroke rehabilitation research is crucial to better understand the pathophysiology, to help predict outcomes, and to indicate adequate rehabilitation therapy [2–4]. Because of the difficulties in measuring unstable free radicals in human samples, the degree of oxidative stress can be determined by measuring the stable end-products of oxidatively modified proteins, lipids, and DNA [5]. The most sensitive biomarker to determine oxidative DNA damage is 8-hydroxy-2′-deoxyguanosine (8-OHdG). Elevated urinary 8-OHdG content released from the brain is closely correlated with the prognosis of stroke [2,6]. Levels of 8-OHdG may reflect the severity of stroke and are thus associated with functional disabilities.

Stroke is a leading cause of death and permanent disability worldwide [4,7,8]. Although medical treatment is available, many stroke survivors still suffer from mild to severe post-stroke disabilities, and their number is growing. Long-term disabilities lead to dependence and to poor quality of life and functional outcomes [3,9]. Recent research emphasizes that rehabilitation exercise is one of the promising approaches to ameliorate motor impairments and poor clinical outcomes in stroke survivors with disabilities [10–12]. Nevertheless, high-intensity physical exercise triggers reactive oxygen species (ROS) production, which then leads to oxidative stress [13]. On one hand, rehabilitation training within a tolerable range is worthwhile to patients to regain their motor function without additional adverse effects on oxidative stress biomarkers.

On the other hand, stroke survivors generally exhibit lower physical capacity and higher oxidative stress than healthy individuals [6,14]. Some studies reported exercise training is beneficial to stroke survivors in regaining the functional outcomes [14,15]. A few studies have advocated the associations between stroke-risk factors and poor functional outcomes [16,17]. However, the association between oxidative stress biomarkers and functional outcomes in stroke survivors undergoing rehabilitation training has not been investigated. Owing to the susceptibility to DNA damage, we assume that facing any physical challenge may affect stress biomarkers in stroke survivors that may lead to improvement or deterioration in their clinical and functional outcomes. Eventually, studies on the potential value of using biomarkers in stroke rehabilitation will gain more insight on how biomarkers could affect functional outcomes in stroke survivors and help to bridge the gap between basic and clinical stroke rehabilitation research. Hence we proposed this exploratory study to emphasize the effects of rehabilitation intervention on 8-OHdG and outcomes, and the changes in these measures were compared before and after rehabilitation. The main objectives of this study were to (1) evaluate the test-retest reliability of 8-OHdG; (2) distinguish the correlation between 8-OHdG and functional outcomes; (3) investigate whether baseline 8-OHdG levels can predict functional outcomes after rehabilitation; and (4) examine the effects of 4 weeks of rehabilitation training on 8-OHdG levels and functional outcomes.

## 2. Results

### 2.1. Characteristics of the Stroke Patients

Table 1 reports the baseline demographic and clinical characteristics of the 61 stroke patients (mean age, 54.6 ± 10.96 years). The mean time from stroke onset was 23 months before the study. The study included 35 patients with right hemispheric lesions and 26 patients with left hemispheric lesions. The stroke type was ischemic in 37 patients and hemorrhagic in 24. Most stroke survivors were nonsmokers (95.1%) or nondrinkers (93.4%) after their stroke onset, and 42.6% took various antioxidant supplements. Nevertheless, 83.6% patients had at least one or more comorbidities, mainly hypertension, diabetes mellitus, heart disease, or osteoarthritis. However, these characteristics showed low and non-significant correlations with baseline 8-OHdG levels (*r* = −0.14 to 0.23, *p* > 0.05). Similarly, the reported stroke types (ischemic and hemorrhagic) were not significantly correlated with functional outcomes in our study (*p* > 0.05).

### 2.2. Test-Retest Reliability of 8-OHdG

**Figure 1.**
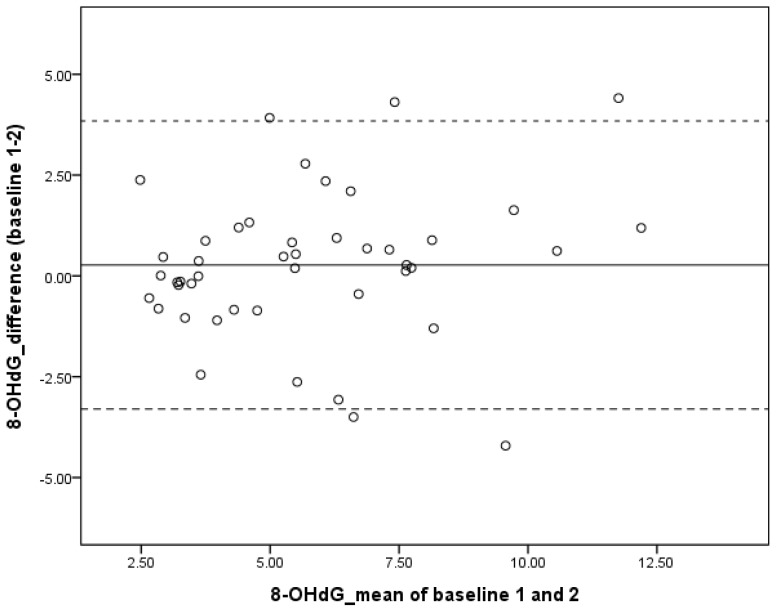
The Bland-Altman plot shows the test-retest reliability of urinary 8-hydroxy-2′-deoxyguanosine (8-OHdG) levels. The reference lines show the mean differences between baseline 1 and baseline 2 assessments of 8-OHdG (solid line: 0.27), and the 95% limits of agreement for the mean difference (dotted lines: −3.30 and 3.84).

The test-retest reliability of 8-OHdG measurements before rehabilitation (*i.e.*, Baselines 1 and 2) was good (intraclass correlation coefficient [ICC] = 0.76 [95% confidence interval limits = 0.60, 0.86]; Table 2). The Bland-Altman plot demonstrated small differences on both baseline measurements (*i.e.*, 0.27 ng/mg creatinine), which indicates a good agreement between two baseline measures. The differences are symmetrically distributed within a 95% confidence interval limit, which further indicates no systematic bias between the measures (Figure 1).

### 2.3. Correlations between 8-OHdG and Functional Outcomes

Before rehabilitation, the FMA and MRC scales had fair and significant correlation with 8-OHdG levels (*r* = −0.38 and *r* = −0.30, respectively; *p* < 0.05). The negative correlation indicates that patients with higher 8-OHdG levels had lower upper-limb motor function and muscle power. No significant correlation with 8-OHdG was found for fatigue (*r* = 0.21) and pain (*r* = 0.13) before rehabilitation; however, positive correlation values for fatigue and pain suggest that patients who rated severe fatigue and pain had higher 8-OHdG levels (Table 3).

We found a fair and significant post-treatment correlation between 8-OHdG and the FMA (*r* = −0.34, *p* < 0.05), as well as between 8-OHdG and pain rating scales (*r* = 0.26, *p* < 0.05; Table 3). The decreased 8-OHdG levels after rehabilitation perhaps partially explains the improved upper-limb motor function and less pain in stroke patients.

### 2.4. Predictive Ability of Baseline 8-OHdG on Post-Stroke Functional Outcomes

The estimated baseline 8-OHdG content had significant correlation with post-treatment functional outcomes of the FMA, MRC, and pain (*r* = −0.34, −0.31, and 0.25, respectively; *p* < 0.05; Table 4). Noticeably, patients with lower 8-OHdG levels before rehabilitation appeared to gain more improvements in motor function and muscle power, and further reported feeling less pain after the 4-week rehabilitation. These findings emphasize the possibility of using 8-OHdG as a biomarker to predict functional outcomes after stroke rehabilitation.

### 2.5. Effect of Rehabilitation on 8-OHdG and Functional Outcomes

Another key finding is that stroke patients demonstrated less DNA damage after the 4-week rehabilitation, which was evidenced by decreased 8-OHdG content (*p* = 0.04; Table 5). Our results reveal that the rehabilitation intervention did not cause any oxidative stress but instead attenuated oxidative DNA damage. In addition, functional outcomes of the FMA, MRC, and pain rating, were significantly improved after rehabilitation compared with the baseline (*p* < 0.05, Table 5). In summary, the decreased 8-OHdG content after rehabilitation significantly correlated with improved functional outcomes, which may imply that patients with less DNA damage are likely to have better improvements in functional outcomes.

## 3. Discussion

To the best of our knowledge, this is the first preliminary report in stroke rehabilitation research to demonstrate urinary 8-OHdG as a reliable biomarker that can be considered a valid predictor of functional outcomes in chronic stroke survivors. The changes in 8-OHdG content were significantly correlated with the scores of upper-limb motor function, muscle power, and perception of pain. Furthermore, significantly decreased 8-OHdG content after a 4-week rehabilitation was correlated with improved functional outcome. Decreased 8-OHdG levels may suggest the possibility of less occurrence of oxidative DNA damage, which partially contributes to improve brain plasticity and functional outcomes. These findings expand the scientific basis of stroke rehabilitation and advance translational rehabilitation research. Similar studies of biomarkers in acute and sub-acute stroke patients may also be useful for early prediction and design the innovative rehabilitation programs.

Reliability examination is crucial for investigating the usefulness of biomarkers in stroke rehabilitation research. Our data suggest that 8-OHdG can be used as a reliable biomarker of oxidative DNA damage in chronic stroke patients. Studies claiming the reliability of 8-OHdG as a biomarker in stroke rehabilitation are still limited; however, the reliability of other biomarkers of oxidative stress have been validated in human samples under different pathologic conditions [18,19]. This study selected subjects, who were chronic stroke patients (onset time > 6 months) and medically stable. This criterion may guide assessments of the identical alterations in 8-OHdG levels that could establish the good reliability and validity of the biomarker. Most of studies on reliability tests were performed in patients, who were medically stable, which may avoid equivocal data and validates the chosen biomarker [20].

Although elevated urinary 8-OHdG levels have been reported in stroke patients [2,6], few studies have depicted the correlation between this biomarker and functional outcomes after stroke rehabilitation. Our study showed a significant correlation between 8-OHdG and impaired functional outcomes and that rehabilitation-induced decreased 8-OHdG is associated with improved upper-limb motor function. A previous study indicated that 8-OHdG levels were higher in acute ischemic stroke patients with poor clinical outcomes than in those with good outcomes [16]. The burst of free radicals, followed by stroke, contribute to neuronal cell death through activated inflammatory biomarkers [2]. Consequently, decreased antioxidant status may set oxidative stress circumstances in the brain, where oxidation of healthy DNA occurs. In this scenario, brain cell are unable to coordinate normal motor function, which may result in motor impairments. The changes in 8-OHdG in our study could be proportionate to the amount of DNA damage and shows its involvement in the progression of poor/good outcomes. However, further molecular studies are encouraged to confirm this phenomenon.

Another key finding is that the biomarker 8-OHdG is a valid predictor of functional outcomes in stroke survivors. The significant association between higher baseline 8-OHdG content and poor outcomes after rehabilitation is convincing evidence of its predictive ability. Changes in 8-OHdG levels after ischemic stroke have been considered as a useful predictor of poor outcomes in patients [16]. Other reports found that hyperglycemia or higher initial blood glucose levels were strong and independent predictors of poor functional outcomes in patients with stroke [17,21]. Indeed, increased oxidative stress resulting from hyperglycemia exacerbates brain damage [7]. In rehabilitation research, monitoring of such biomarkers as key predictors is not only helpful for understanding the molecular and cellular mechanisms responsible for clinical response [2] but also worthwhile for setting the treatment goals, starting early rehabilitation, and informing patients about their prognosis [22,23].

This is the first preliminary study to demonstrate the possible beneficial effects of rehabilitation intervention on oxidative DNA damage and functional outcomes in stroke survivors. The changes in 8-OHdG levels before and after rehabilitation were statistically compared to determine the rehabilitation effect. We found considerably decreased 8-OHdG levels after a 4-week rehabilitation intervention. Because elevated urinary 8-OHdG is considered a marker of brain injury [6], higher 8-OHdG levels before rehabilitation indicate more DNA damage in patients. Unlike proteins and lipids, DNA is able to repair oxidatively modified bases through its own DNA repair system [24,25]. Decreased DNA damage after rehabilitation in our study could be due to the increased antioxidant capacity or decreased ROS production, or both. Previous studies showed that regular exercise training can improve the antioxidant status and decrease 8-OHdG levels in older adults [25,26]. Therefore, our findings suggest that 4 weeks rehabilitation training may facilitate oxidation of less DNA and simultaneously contribute to improving brain physiology and regaining motor function. One of our previous studies also demonstrated that regular robot-assisted therapy may be suitable for patients with chronic upper-limb disabilities without causing oxidative stress [27]. In addition, the rehabilitation intervention in this study did not produce any additional oxidative stress but instead attenuated the DNA damage. This result indicates that the tolerance of training intensity may be suitable for practical use in stroke rehabilitation units. Although decreased 8-OHdG levels may partially explained the improved functional outcomes, several other biochemical parameters involved in this process need to be addressed in future studies.

The results obtained on upper-limb motor function and reduced pain after rehabilitation has confirmed the close association with decreased DNA damage. Regardless of the rehabilitation, our findings of decreased 8-OHdG levels and increased clinical outcomes are in agreement with a previous study [16]. A noteworthy finding is that patients with lower baseline 8-OHdG levels tended to gain more improvements in motor function, which implies the necessity of an 8-OHdG evaluation in patients receiving rehabilitation training. Furthermore, patients reported no increased adverse effects (*i.e.*, pain and fatigue) during the rehabilitation intervention. We also observed that patients with severe pain had higher baseline 8-OHdG levels and that those with lower 8-OHdG levels before treatment reported reduced pain scores after treatment. Reasonable relationships between 8-OHdG and pain were observed in this study, but the role of DNA damage in pain of stroke survivors needs further investigation.

### Limitations and Further Suggestions

To establish a strong correlation between biomarkers of oxidative stress and functional outcomes, further evaluation of antioxidant status and other oxidative stress markers (e.g., protein carbonyls and isoprostanes, widely used protein oxidation and lipid peroxidation markers) in blood or urine samples is necessary. In addition, advanced research techniques, such as magnetic resonance imaging, could provide the additional direct evidence to explain the vascular remodeling, cerebral angiogenesis, and neural plasticity that are associated with the decreased oxidative stress biomarkers after rehabilitation intervention in stroke survivors. Lack of a control arm and other clinical scales, including the modified Rankin Scale and Barthel Index, are the additional limitations in our study, which are considerable outcomes to include in future rehabilitation studies.

## 4. Experimental Section

### 4.1. Participants

In this secondary analysis study, the effects of stroke rehabilitation programs were investigated in 61 stroke patients. All patients were carefully screened for the following inclusion criteria: (1) a unilateral ischemic or hemorrhagic stroke; (2) able to follow the study instructions and perform study tasks; (3) no upper-limb fractures within the last 3 months or painful arthritis or injuries of the joints; (4) no acute inflammatory or infectious disease; (5) no participation in any experimental rehabilitation or drug studies during the study period; (6) medically stable; and (7) willing to provide written informed consent. Institutional review board approval was obtained from the participating hospitals, and written consent was obtained from each patient before inclusion. Stroke type, smoking and drinking behavior, and intake of antioxidant supplements were recorded along with anthropometric variables. Participants were asked to keep the same intake of antioxidant supplements (e.g., vitamin E, vitamin C, or β-carotene) during the study period.

### 4.2. Rehabilitation Intervention

Patients received their rehabilitation programs, including robot-assisted therapy, combined rehabilitation therapy, and customary rehabilitation, for 20 training sessions during the 4-week study period. Each training session comprised 90 to 105 min/day, 5 days/week. The rehabilitation intervention was provided by licensed occupational therapists experienced in the administration of training protocols. Functional outcomes were administered to the patients before and after the rehabilitation intervention by a blinded rater who was well trained to appropriately conduct these functional assessments.

### 4.3. Urine Sample Collection

A urine sample (10 mL) was collected from the 61 patients before (*i.e.*, baseline 1) and after rehabilitation (*i.e.*, post-treatment). To determine the test-rest reliability of 8-OHdG, baseline sample collections (before rehabilitation) were done twice (*i.e.*, baselines 1 and 2) with a 3-day interval from 45 of the 61 patients. The collected samples were properly labeled and stored at −20 °C until the 8-OHdG analysis.

### 4.4. Determination of 8-OHdG Levels by LC-MS/MS

Highly-sensitive liquid chromatography with tandem mass spectrometry (LC-MS/MS) was used to analyze the urinary 8-OHdG levels [28]. Briefly, a urine sample (500 μL) was diluted with water (500 μL), followed by the addition of 20 μL ^15^*N*_5_-8-OHdG solution (42.6 ng/mL) as the internal standard. The prepared sample (10 μL) was injected into the ultra-performance LC-MS/MS system, which was equipped with an Accela autosampler and micropump (Thermo Fisher Scientific, Waltham, MA, USA), an L-2100 pump (Hitachi High-Technologies, Tokyo, Japan) and two reverse-phase columns (4.6 × 33 mm, 5 μm; Inertsil ODS-3 (GL Sciences, Tokyo, Japan) and 2.1 × 100 mm, 3 μm; Atlantis T3 (Waters Corporation, Milford, MA, USA). Creatinine levels were used to correct for variations in urine concentration.

### 4.5. Functional Outcomes

#### 4.5.1. Fugl-Meyer Assessment (FMA)

The 33-item FMA was used to determine the upper-limb motor impairments of the stroke patients [29]. The FMA upper-limb subscale is relevant to the movement, reflexes, and coordination of the shoulder, elbow, forearm, wrist, and hand. The items are scored on a 3-point scale: 0, cannot perform; 1, performs partially; and 2, performs fully. The maximum score of the FMA is 66, indicating normal motor performance. The reliability, validity, and responsiveness of the FMA have been well demonstrated in stroke patients [9,30].

#### 4.5.2. Medical Research Council (MRC) Scale

Muscle power of affected arm was examined by the MRC scale, a reliable assessment in stroke patients, with a score range from 0 to 5 [31]. Grade 0 on the MRC scale indicates no contraction; 1, flicker or trace contraction; 2, active movement, with gravity eliminated; 3, active movement against gravity; 4, active movement against gravity and resistance; and 5, normal power.

#### 4.5.3. Assessment of Pain and Fatigue

Pain and fatigue, two common complications after stroke that affect the patient’s well-being were also measured [32,33]. Severity of pain and fatigue were measured on the first and final day of the treatment by using a single item of the Numerical Rating Scale [34]. All patients were asked to rate their pain and fatigue feeling severity on a scale of 0 to 10, with 0 indicating no pain/no fatigue and 10 representing unbearable pain/exhaustion.

### 4.6. Statistical Analysis

The 8-OHdG data from the two baseline measurements were used to examine the test-retest reliability by the ICC. The ICC is considered high at >0.75, moderate at 0.4 to 0.74, and poor at <0.40 [35,36]. The Bland-Altman plot was constructed to examine the agreement between two baseline measurements by plotting the differences between the measurements (*y*-axis) against the mean values (*x*-axis) [37]. The 95% confidence interval limits of agreement on the plot were calculated by mean difference ±1.96 standard deviation. A smaller interval between the 95% limits represents better agreement.

The Pearson correlation coefficient (*r*) was used to determine the associations between 8-OHdG and functional outcomes. An *r* value between 0 and 0.25 indicates low, between 0.25 and 0.5 indicates fair, between 0.5 and 0.75 indicates moderate to good, and greater than 0.75 indicates good to excellent correlations [20].

The changes in 8-OHdG levels and functional outcomes after rehabilitation were examined with the paired *t* test. Statistical analyses were performed with SPSS 19 software (SPSS Inc., Chicago, IL, USA). Values are expressed as mean ± standard deviation, and the significance level was set at *p* < 0.05.

## 5. Conclusions

Our study has delineated for the first time the correlation between 8-OHdG and functional outcomes in stroke rehabilitation. The findings demonstrated that higher baseline 8-OHdG content represents poor functional outcomes, whereas lower 8-OHdG after rehabilitation resulted in improved functional outcomes. The results indicate that 8-OHdG can be used as a reliable and valuable biomarker to predict functional outcomes in stroke rehabilitation. Our study further demonstrates the promising prognostic values of 8-OHdG and its associations with functional outcomes in stroke survivors. In addition to rehabilitation therapy, future studies may consider examining the effects of complementing rehabilitation with the use of free radical-scavenging drugs in patients with stroke.

## Figures and Tables

**Table 1.** Demographic and clinical characteristics of the patients with stroke (*N* = 61).

Characteristics	Value

	Mean ± SD or No. (%)
Age, years	54.60 ± 10.96

Time after stroke, months	23.46 ± 14.00

Sex	

Male	39 (63.9)
Female	22 (36.1)

Side of stroke	

Right	35 (57.4)
Left	26 (42.6)

Stroke type	

Ischemic	37 (60.7)
Hemorrhagic	24 (39.3)

Smoker	3 (4.9)

Drinker	4 (6.6)

Antioxidants intake	26 (42.6)

Comorbidities	

Hypertension	41
Diabetes mellitus	23
Heat disease	18
Osteoarthritis	5
Hyperlipidemia	2
Gout	1
Epilepsy	1
Thalassemia	1

**Table 2.** The test-retest reliability of 8-OHdG from the two baseline measures in patients with stroke (*N* = 45). Abbreviations: 8-OHdG, 8-hydroxy-2′-deoxyguanosine; CI, confidence interval; ICC, intraclass correlation coefficient; SD, standard deviation.

Variable	8-OHdG (ng/mg creatinine) (Mean ± SD)	Mean difference	ICC (95% CI)
Baseline 1	5.87 ± 2.77		
Baseline 2	5.60 ± 2.47	0.27	0.76 (0.60–0.86)

**Table 3.** Correlations between 8-OHdG levels and functional outcomes before and after rehabilitation intervention (*N* = 61). Abbreviations: 8-OHdG, 8-hydroxy-2′-deoxyguanosine; CI, confidence interval; FMA, Fugl-Meyer Assessment; and MRC, Medical Research Council scale.

Functional outcome	Pearson *r* (95% CI)	

	Pre-treatment	Post-treatment
FMA	−0.38* (−0.58 to −0.14)	−0.34* (−0.55 to −0.10)
MRC	−0.30* (−0.51 to −0.05)	−0.19 (−0.42 to 0.06)
Fatigue	0.21 (−0.04 to 0.44)	0.18 (−0.08 to 0.41)
Pain	0.13 (−0.13 to 0.37)	0.26* (0.10 to 0.48)

* *p* < 0.05.

**Table 4.** Predictive ability of pre-treatment 8-OHdG levels on post-treatment functional outcomes in stroke patients (*N* = 61). Abbreviations: 8-OHdG, 8-hydroxy-2′-deoxyguanosine; CI, confidence interval; FMA, Fugl-Meyer Assessment; and MRC, Medical Research Council scale.

Functional outcome	Pearson *r* (95% CI)
FMA	−0.34* (−0.55 to −0.10)
MRC	−0.31* (−0.52 to −0.06)
Fatigue	0.21 (−0.04 to 0.44)
Pain	0.25* (0 to 0.47)

* *p* < 0.05.

**Table 5.** Effects of 4-week rehabilitation intervention on 8-OHdG levels and functional outcomes in patients with stroke (*N* = 61). Abbreviations: 8-OHdG, 8-hydroxy-2′-deoxyguanosine; FMA, Fugl-Meyer Assessment; MRC, Medical Research Council scale; SD, standard deviation.

Measurement (units)	Pre-treatment (Mean ± SD)	Post-treatment (Mean ± SD)	*p* value
8-OHdG (ng/mg creatinine)	5.02 ± 2.72	4.46 ± 2.37	0.04*
FMA (0–66)	43.75 ± 8.96	47.70 ± 9.13	<0.01*
MRC (0–5)	3.57 ± 0.64	3.79 ± 0.64	<0.01*
Fatigue (0–10)	2.32 ± 2.47	2.16 ± 2.34	0.53
Pain (0–10)	1.82 ± 2.56	1.08 ± 1.83	0.02*

* *p* < 0.05.
